# Structural Basis for the Functional Diversity of Centrins: A Focus on Calcium Sensing Properties and Target Recognition

**DOI:** 10.3390/ijms222212173

**Published:** 2021-11-10

**Authors:** Marco Pedretti, Luca Bombardi, Carolina Conter, Filippo Favretto, Paola Dominici, Alessandra Astegno

**Affiliations:** Department of Biotechnology, University of Verona, Strada Le Grazie 15, 37134 Verona, Italy; marco.pedretti@univr.it (M.P.); luca.bombardi@univr.it (L.B.); carolina.conter@univr.it (C.C.); filippo.favretto@univr.it (F.F.); paola.dominici@univr.it (P.D.)

**Keywords:** centrin, EF-hand, calcium signaling, calcium binding protein, protein interaction

## Abstract

Centrins are a family of small, EF hand-containing proteins that are found in all eukaryotes and are often complexed with centrosome-related structures. Since their discovery, centrins have attracted increasing interest due to their multiple, diverse cellular functions. Centrins are similar to calmodulin (CaM) in size, structure and domain organization, although in contrast to CaM, the majority of centrins possess at least one calcium (Ca^2+^) binding site that is non-functional, thus displaying large variance in Ca^2+^ sensing abilities that could support their functional versatility. In this review, we summarize current knowledge on centrins from both biophysical and structural perspectives with an emphasis on centrin-target interactions. In-depth analysis of the Ca^2+^ sensing properties of centrins and structures of centrins complexed with target proteins can provide useful insight into the mechanisms of the different functions of centrins and how these proteins contribute to the complexity of the Ca^2+^ signaling cascade. Moreover, it can help to better understand the functional redundancy of centrin isoforms and centrin-binding proteins.

## 1. Overview of Centrins

Centrins are a family of proteins that contain EF-hands that have functional similarity with calmodulins (CaM), and like CaM, have a number of unrelated functions. Centrins are ubiquitous in eukaryotes and for the most part are associated with microtubule organizing center structures (MTOCs) or cytoplasmic eukaryotic organelles that have a role in nucleation and spatial organization of microtubules [1,2,3].

The first centrin to be identified was a major constituent of striated flagellar rootlets in *Tetraselmis striata* [4], a green algae, where it functions in calcium (Ca^2+^)-dependent contractions. Molecular analysis of centrin orthologues in mammals [5,6,7] showed that it is associated with centrosomes and established that it is highly conserved. The orthologue centrin in *Saccharomyces cerevisiae* (CDC31) was found to be localized in the half-bridge of the spindle pole body (SPB) which is a microtubule-organizing center analogous to the centrosome in mammals [8,9]. Centrin genes have been found in all eukaryotic kingdoms [2,5,6,9,10,11,12,13,14,15]. While centrins play a role in the function of centrosomes, the vast majority (≈90%) do not appear to be associated with centrosomes [16]. It has been suggested that such widespread distribution in cells is probably related to different functions in the cytoplasm and nucleus. Indeed, centrins are believed to be involved in processes such as repair of DNA, duplication of centrosomes, and nuclear export of mRNA, as well as in protein degradation and signal transduction [17,18,19,20,21,22,23,24,25,26,27,28].

Considering the number of centrin genes in different organisms, lower eukaryotes such as *Saccharomyces cerevisiae* and *Chlamydomonas reinhardtii* possess one gene for centrin (CDC31 and CrCEN, respectively), while higher eukaryotes normally have several isoforms due to gene duplication or retrotransposition events involving mRNA [29]. In humans, there are three centrin genes (HsCEN1 to 3) [5,6,12,30]. Mice have an additional isoform that can chromosomally integrate as shown by the cases of murine centrin 1 (MmCEN1) [31] and centrin 4 (MmCEN4) [32]. In other species, the number of centrin genes may be much higher: the ciliate *Paramecium caudatum* has up to 22, while the parabasalia *Trichomonas vaginalis* has 24 [33]. Four centrin-like proteins have been described in *Plasmodium falciparum* (PfCEN1 to 4) [34], compared to five in the parasite *Trypanosoma brucei* (TbCEN1 to 5) and three in *Toxoplasma gondii* (TgCEN1 to 3) [15,35,36]. This indicates that centrins have undergone considerable and complex diversification in eukaryotes, involving multiple duplication events and gene losses.

Data from proteomic and clustal analyses of amino acid sequences of centrins in various species suggested the existence of at least two divergent protein subfamilies in the centrin family [12,37]. In the largest subfamily, CrCEN is the prototype that also includes the human centrins HsCEN1 and HsCEN2, while HsCEN3 and yeast centrin CDC31 comprise a distinct subfamily. Aubusson-Fleury et al. recently updated previous phylogenetic analyses by adding ciliate centrins, defining five main functional families [33]. The first two are formed by classical basal body associated centrins, CEN2/CrCEN and CEN3/CDC31, while ciliary centrins form a new family [38]. The fourth family of centrins contains a centrin required for the ciliary voltage gated Ca^2+^ channel (VGCC centrin) from *Paramecium caudatum* [39] along with a centrin ortholog from *Paramecium tetraurelia*, which has a similar function. A large fifth family contains centrins that are located in contractile filaments (ICL centrins) and are needed for contractile network assembly [33,40].

## 2. Centrins Have Large Differences in Their Ability to Sense Ca^2+^

**Figure 1:**
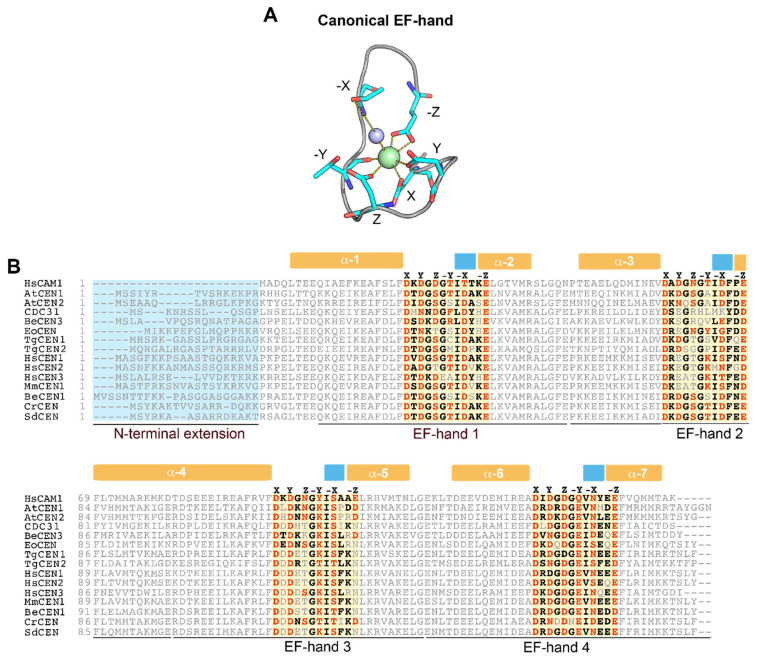
Overview of the EF-hand Ca^2+^ binding domains in different centrins. (**A**) Ca^2+^ coordination by the canonical EF-hand (PDB: 1CLL). The Ca^2+^ ion is coordinated in a pentagonal bipyramidal configuration by ligands indicated by their position in the coordination geometry (X, Y, Z, −X, −Y and −Z). NH groups of coordinating amino acids are indicated in dark blue, oxygen atoms in red, the Ca^2+^ ion in green and the coordinating water molecule in violet. (**B**) Protein sequence alignment of centrins from different organisms. The N-terminal extension (light blue box) and the central 12 residues in the EF-hand domains (orange boxes) are highlighted. Within the EF-hands, Ca^2+^ chelating residues are represented in orange while the other most common residues are represented in black. Secondary structural elements derived from the 3D structure of human CaM (PDB: 1CLL), α-helices (orange) and β-sheets (light blue) are displayed on the top of the alignment.

Centrins are acidic proteins that are around 170 amino acids long. Similar to CaM, centrins have two lobes, the C-terminal domain and N-terminal domain, which are separated by a linker region, shaping like a dumbbell [41,42,43]. The lobes are structurally independent, and each consists of two 29-amino acid helix-loop-helix structures, called EF-hand motifs, that can bind Ca^2+^. The central 12 residues in the EF-hand have the ability to form a turn-loop structure that coordinates one Ca^2+^ ion (Figure 1A) [44]. This takes place through a pentagonal bipyramidal configuration involving a number of residues: carboxylate oxygens from 1 (+X), 3 (+Y), 5 (+Z) and 12 (−Z), carbonyl oxygen from 7 (−Y), and a bridged water at residue 9 (−X). The loop generally contains three aspartic acid residues that bind Ca^2+^ and form the DxDxDG motif. Together with a Gly residue at position 6, which permits the loop to wrap around Ca^2+^, this allows for high affinity binding of the ion [44]. In most centrins, residue 12 is glutamate, which provides bidentate chelation, even if substitution of glutamate with aspartic acid is not uncommon. This is worthy of note since substitution with aspartate decreases binding selectivity of Ca^2+^ to favor binding of Mg^2+^ ions [44,45].

Phylogenetic analyses have suggested that EF-hand domains arose from two rounds of duplications of an ancestral EF-hand [46]. Centrins possess four EF-hand domains that are highly coherent with the responsive actions to Ca^2+^ in which the proteins were first discovered. Nonetheless, over time, in centrins, some EF-hand motifs have lost the ability to bind Ca^2+^. In this regard, predictions of function [47] have suggested that several motifs are probably non-functional, considering deletions in the EF-binding loop or the absence of key residues for coordination. Thus, while all centrins possess four EF-hand motifs, there are large differences in their ability to bind Ca^2+^. This supposition has been confirmed by biophysical analyses, thus adding weight to the hypothesis that differences in the amino acid sequences of EF-hands are responsible for protein functional versatility (Table 1 and Figure 1B).

For example, HsCEN2 has two functional Ca^2+^ binding sites in EF-3 and EF-4 [43,48,49,50]. Notably, EF-3 has an Asn at position 12, which is likely responsible for the low affinity of this site. HsCEN3 has one high affinity mixed Ca^2+^/Mg^2+^ binding and two Ca^2+^-specific sites of low affinity. EF-3 is inactive due to the Asn for Glu change at position 12. In addition, EF-1 has a Glu in position 6, and EF-2 should have low affinity binding given the existence of a Thr in position 5. In contrast, MmCEN1 possesses four EF-hands that all bind Ca^2+^ even if with apparently different affinities since, as shown by the structure, there are fewer ligands involved in the Ca^2+^ binding in EF-1 and EF-2 compared to EF-3 and 4 [51]. Centrin from yeast binds three Ca^2+^ ions, one with high affinity at EF-1 and two with low-affinity at EF-3 and EF-4 [19]. At position 12, EF-3 has an Asn instead of Glu. The EF-2 site appears to be inactive, given that it has an Arg in position 5, a His at position 6, and an Asp at the critical position 12. Centrins from green algae *Scherffelia dubia* (SdCEN) and CrCEN have two high affinity sites in the N-lobe and one moderate affinity in the C-lobe (likely the EF-4) [52]; in CrCEN EF-3 has decreased ability to bind Ca^2+^ (affinity outside the physiological range) considering the Asp for Glu substitution at position 12, while EF-3 of SdCEN is not functional due to Asn for Glu replacement at the same position. Centrin 2 from *Arabidopsis thaliana* (AtCEN2) has four sites that are specific for Ca^2+^ binding and, interestingly, its N-terminal domain contains the sites with higher affinity [22]. Substantial variability among centrins has also been noted in protists. For example, TbCEN4 binds Ca^2+^ with high affinity via EF-3 and EF-4 [14]. TgCEN1 is able to bind two Ca^2+^ ions via EF-1 and EF-2 with high affinity, and TgCEN2 can bind only one Ca^2+^ ion with low affinity through EF-1 [53]. Unusually, neither TgCEN1 nor TgCEN2 EF-3 fit the consensus for an Asn in position 12. Moreover, in both centrins, EF-4 cannot bind Ca^2+^, even if both have an EF-hand consensus sequence. This finding reflects that predicted and experimentally measured binding can differ greatly as previously seen with other Ca^2+^ binding proteins [54,55,56,57].

Given the above, it appears clear that centrin proteins display no consistent evolutionary conservation of Ca^2+^ sites. In some proteins, the EF-hand motifs do not bind Ca^2+^, and as such may be pseudo-EF-hand motifs. Additionally, centrins have distinctly different Ca^2+^ binding properties compared to CaM, which can be considered evidence of their functional specialization. CaM, for example, has four high affinity Ca^2+^-binding sites [58,59], allowing for efficient response to changes in intracellular Ca^2+^. In contrast, centrins have a much wider range of affinity (Table 1).

The selective binding of Ca^2+^ to centrins through EF-hands likely leads to structural rearrangement of α-helices and brings about conformational changes from closed to open, with exposure of a hydrophobic surface that can interact with other proteins involved in cellular signaling. Although the global folding pattern of centrins is somewhat similar to CaMs, centrins have a variable, non-structured positively charged region of 20–25 residues in the N-terminal (Figure 1B). However, at present, the role of this extension in the N-terminal remains unclear. Studies on centrins have suggested that Ca^2+^-induced polymerization appears to depend on this amino-terminal domain [60]. In both HsCEN2 and TgCEN1, this N-terminal extension is needed for self-assembly induced by Ca^2+^ [60,61,62,63].

**Table 1:** Ca^2+^ binding properties of centrins.

Organism	Centrin	UniProt Code	Experimental Ca^2+^-Binding Sites ^a^	Ca^2+^ Affinity ^b^	Refs
Chlamydomonas reinhardtii	CrCEN	P05434	4	K_d1,2N_ = 1.2 ± 0.1 × 10^−6^ M K_d3C_ = 2 ± 2 × 10^−5^ M K_d4C_ = 3 ± 2 × 10^−3^ M	[64,65,66]
Scherffelia dubia	SdCEN	Q06827	3	K_a1N_ = 2.6 × 10^5^ M^−1^ K_a2N_ = 4.3 × 10^5^ M^−1^ K_a3C_ = 1.1 × 10^5^ M^−1^	[52]
Homo sapiens	HsCEN1	Q12798	4	K_a1,2_ = 4.26 × 10^5^ ± 9.5 × 10^4^ M^−1^ K_a3,4_ = 2.73 × 10^4^ ± 2.7 × 10^3^ M^−1^	[67,68]
Homo sapiens	HsCEN2	P41208	2	K_a(EF-3)_ = 8.1 × 10^3^ M^−1^ K_a(EF-4)_ = 1.5 × 10^5^ M^−1^	[43,48,49,50,62]
Homo sapiens	HsCEN3	O15182	3	K_a1N_ = 3.3 × 10^5^ M^−1^ K_a2_ = 7.0 × 10^3^ M^−1^ K_a3_ = 7.5 × 10^3^ M^−1^	[69]
Saccharomyces cerevisiae	CDC31	P06704	3	K_a(EF-1)_ = 3.0 × 10^6^ M^−1^ K_a2C_ = 2.4 × 10^4^ M^−1^ K_a3C_ = 3.5 × 10^4^ M^−1^	[19,70]
Mus musculus	MmCEN1	P41209	4	K_a1_ = 5.23 × 10^5^ M^−1^ K_a2_ = 3.11 × 10^3^ M^−1^ K_a3_ = 2.31 × 10^5^ M^−1^ K_a4_ = 1.59 × 10^4^ M^−1^	[51]
Arabidopsis thaliana	AtCEN2	O23184	4	K_a(EF-1)_ = 2.9 × 10^5^ ± 7.1 × 10^4^ M^−1^ K_a(EF-2)_ = 4.1 × 10^5^ ± 6.8 × 10^4^ M^−1^ K_a(EF-3)_ = 1.4 × 10^4^ ± 3.8 × 10^3^ M^−1^ K_a(EF-4)_ = 3.7 × 10^3^ ± 0.8 × 10^3^ M^−1^	[22]
Toxoplasma gondii	TgCEN1	A0A125YHX7	2	K_a(EF-1)_ = 4.8 × 10^5^ ± 6.1 × 10^3^ M^−1^ K_a(EF-2)_ = 3.9 × 10^4^ ± 4.5 × 10^3^ M^−1^	[53]
Toxoplasma gondii	TgCEN2	A0A125YZN2	1	K_a(EF-1)_ = 1.6 × 10^4^ ± 1.5 × 10^3^ M^−1^	[53]
Trypanosoma brucei	TbCEN4	A0A3L6L623	2	K_a(EF-3)_ = 3.18 × 10^5^ ± 4.63 × 10^4^ M^−1^ K_a(EF-4)_ = 2.63 × 10^4^ ± 4.37 × 10^3^ M^−1^	[14]
Trypanosoma brucei	TbCEN5	Q382E7	2	K_d1,2_ = 4.8 µM	[71]
Euplotes octocarinatus	EoCEN	Q9XZV2	4	K_a1,2_ = 1.12 ± 0.04 × 10^3^ M^−1^ K_a(EF__-4)_ = 6.82 ± 0.33 × 10^5^ M^−1^	[13,72]
Blastocladiella emersonii	BeCEN1	Q4F6W6	4	K_d1_ = 6.06 ± 2.26 µM K_d2_ = 7.50 ± 0.44 µM K_d3_ = 75.20 ± 28.3 µM K_d4_ = 9.35 ± 0.93 µM	[73]
Blastocladiella emersonii	BeCEN3	Q4F6W5	4	K_d1_ = 2.45 ± 0.04 µM K_d2_ = 18.50 ± 0.86 µM K_d3_ = 2.11 ± 0.38 µM K_d4_ = 38.1 ± 7.46 µM	[73]

^a^ Number of functional Ca^2+^-binding sites as experimentally measured by isothermal titration calorimetry (ITC), nuclear magnetic resonance (NMR) or flow dialysis analysis. ^b^ K_a_ = equilibrium association constant; K_d_ = dissociation constant. K_d_ is the inverse of the equilibrium association constant, K_a_, (i.e., K_d_ = 1/K_a_). N = N-terminal domain. C = C-terminal domain. Where the EF-hand site is not specified, binding affinities refer to the affinity for the first, second, third and fourth Ca^2+^ bound, not to the affinity of the individual sites.

## 3. Functional Diversity and Specialization of Centrins

Similar to other members of the CaM subfamily, centrins likely act as Ca^2+^ sensors, interacting with target proteins to regulate specific cellular activities. However, some centrins can also bind to their target independently of Ca^2+^ [19,21,28,52,70]. The current five targets for centrins have been well established: XPC (xeroderma pigmentosum group C protein) [27]; SFI1 (suppressor of fermentation-induced loss of stress resistance protein 1) [74]; SAC3 (suppressor of actin) [20]; KAR1 (karyogamy protein) [9]; and transducin [23]. Notwithstanding, new targets are being discovered in many organisms, which are involved in a large variety of cellular processes (Table 2).

Since centrins recognize specific proteins, both centrins and their targets have features that allow for their mutual recognition. Some authors have hypothesized that this is related to the intrinsic disorder of the target sites that subsequently gain ordered structure following the binding of centrin [19,21,22,52,53,66,75,76]. It has been shown that the hydrophobic pocket of centrin is able to bind its target using a hydrophobic triad, namely W^1^xxL^4^xxxL^8^ (1–4–8 motif) [19,21,76]. Interestingly, there are two orientations of the centrin-binding motif: W^1^xxL^4^xxxL^8^ and L^8^xxxL^4^xxW^1^ (8-4-1) [21,70]. In these binding motifs, the positions 1 and 4 are always hydrophobic residues, position 8 is less conserved. Studies on HsCEN2 bound to XPC have noted the importance of W1 as major determinant for anchoring and is located within a hydrophobic site in the C-lobe of the centrin [43,49,76].

Crystal [20,43,49,70] and NMR [50,77,78,79] structures of centrins complexed with target sequences (i.e., XPC, SFI1, SAC3 or KAR1) have been solved (Table 2). The pocket that binds the target is situated in the C-terminal portion of centrin, and in human centrin residue F113 is a key player in target binding. In the target, the W residue of the triad lies within the binding cavity in proximity of centrin F113. The overall structure of the N- and C-terminal domains is analogous in HsCEN2 and CDC31 when the target is present: the N-terminal domain remains in a closed conformation, whereas the EF-hand helices run anti-parallel; the C-terminal domain has perpendicular helices and has an open conformation. Of note, unlike CaM and troponin C, the C-terminal domain of centrins is also preferentially in an open conformation in the absence of Ca^2+^ [50,64]. Thus, HsCEN2 and CDC31 have the ability to bind targets in their C-terminal via a hydrophobic pocket, even independently of Ca^2+^ [19,21,49,52,64,69,76]. Notwithstanding, for some centrins, target binding through the N-terminal domain has also been documented [52,63].

Moreover, as for CaM, the Ca^2+^ affinity of some centrins considerably increases upon interaction with target proteins [21,66]. Given the above, constitutive binding between centrin and its targets, even at the low Ca^2+^ level of a resting cell, has been hypothesized. Such an example is the binding of centrin CDC31 to KAR1 from yeast [80]. However, in the absence of more in-depth structural comparison among different centrin-target complexes, a generalized role for Ca^2+^ in formation of such complexes cannot be ruled out. Moreover, the majority of structural and biophysical investigations so far have not studied full-length target proteins, but rather only smaller peptides containing the binding region of centrin.

Overall, Ca^2+^ signaling is complex, and greater understanding about crosstalk between pathways and different classes of sensors is needed. In this regard, the numerosity of centrin isoforms and centrin targets highlights this complexity. The use of one or another signaling pathway may be related to several factors, such as the levels of the individual centrin protein, and its differential expression in differentiation and development, as well as to variations in the Ca^2+^ signal, affinity of the EF-hand for Ca^2+^ and phosphorylation status [4,81,82,83,84,85,86].

**Table 2:** Centrin targets.

Centrin	Identified Target	Complex Localization	Function/Pathway	Centrin Binding Motif of Targets	Target Binding Affinity ^a^	PDB CODE	Refs
HsCEN2	XPC	Nucleus	NER	847-NWKLLAKGLLIRERLKR-863	with Ca^2+^, K_a_ = 170 ± 30 × 10^6^ M^−1^ without Ca^2+^, K_a_ = 8 ± 1 × 10^6^ M^−1^	2GGM, 2OBH, 2A4J	[17,43,49,76,77,87]
HsCEN2	XPA	Nucleus	NER	N/A ^b^	N/A	N/A	[87,88]
CDC31	RAD4	Nucleus	DNA repair and protein degradation	N/A	N/A	N/A	[28]
AtCEN2	AtRAD4	Nucleus	NER	756-EAQAASRWYQLLSSILTR-773	with Ca^2+^, K_d_ = 54 ± 14 nM without Ca^2+^, K_d_ = 8 ± 1 µM	N/A	[18,22]
HsCEN1	HsSFI1 (R18) ^c^	Basal body/ centrosome	Centrosome duplication	670-REVAARESQHNRQLLRGALRRWK-692	with Ca^2+^, K_a_ = 12.03 × 10^5^ M^−1^ without Ca^2+^, K_a_ = 0.03 × 10^5^ M^−1^	N/A	[29,89]
HsCEN2	HsSFI1(R17)	Basal body/ centrosome	Centrosome duplication	641-RADLHHQHSVLHRALQAWVT-660	with Ca^2+^, K_a_ = 6.5 ± 0.9 × 10^6^ M^−1^ without Ca^2+^, K_a_ = 0.25 ± 0.02 × 10^6^ M^−1^	2K2I	[29,78,90]
CDC31	ScSFI1 (R18)	SPB	SPB duplication	680- IQAISKRNYQLEKMVLKKFR -699	with Ca^2+^, K_a_ = 1 ± 0.03 × 10^7^ M^−1^ without Ca^2+^, K_a_ = 2.4 ± 0.13 × 10^5^ M^−1^	2GV5, 2DOQ	[19,70,74,91]
CDC31	ScSFI1 (R19)	SPB	SPB duplication	710- ELADEVREEFVLVKTFYIWK -729	with Ca^2+^, K_a_ = 3.5 ± 0.29 × 10^7^ M^−1^ without Ca^2+^, K_a_ = 1.9 ± 0.3 × 10^5^ M^−1^	N/A	[19,70,74,91]
HsCEN2 HsCEN3	GANP	Nuclear pore	mRNA export	1225-IFQTAKETLQELQCFCKYLQRWR-1247	N/A	N/A	[20,25,92]
CDC31	SAC3	Nuclear pore	mRNA export	797-KFFEKWQASYSQAKKNRI-814	with Ca^2+^, K_a_ = 2.2 ± 0.2 × 10^7^ M^−1^ without Ca^2+^, K_a_ = 1.5 ± 0.11 × 10^6^ M^−1^	3FWB, 3FWC, 4MBE	[19,20,24,93,94]
AtCEN1 AtCEN2	SAC3B	Nuclear pore	mRNA export	1050-AKAKLKLIIRLWKRWSSRQSELRERR-1075	with Ca^2+^, K_a_ = 1.5 x 10^6^ ± 5.5 × 10^5^ M^−1^ without Ca^2+^, K_a_ = 4.1 x 10^5^ ± 8.3 × 10^4^ M^−1^	N/A	[21,95,96]
CDC31	KAR1	SPB	SPB duplication	237-KKRELIESKWHRLLFHDKK-255	with Ca^2+^, K_a_ = 2.3 ± 0.15 × 10^7^ M^−1^ without Ca^2+^, K_a_ = 4.4 ± 0.1 × 10^5^ M^−1^	N/A	[8,19,80,97]
HsCEN1	Transducin β	Photoreceptor Connecting Cilium	Phototransduction	325-MAVATGSWDSFLKIWN-340	with Ca^2+^, K_a_ = 0.17 ± 0.12 × 10^6^ M^−1^	N/A	[23,29,84]
HsCEN2	POC5	Basal body/ centrosome	Centriole elongation	156-LQKMENVLDLWSSGLKTN-173 245-KIELMRTFFHWRIGHVRA-262 278-RTLLKKVWKVWRSVVQKQ-295	N/A	N/A	[98,99]
HsCEN2	PRP40A	Nucleus	Pre-mRNA splicing	524-KQLRKRNWEALKNILDNMANVTYSTTWSEAQQY-556	with Ca^2+^, K_a_ = 3.6 x ± 0.4 × 10^6^ M^−1^	N/A	[75]
HsCEN2	NUP107-160	Nuclear pore	mRNA and protein nuclear export	N/A	N/A	N/A	[25]
HsCEN2	MPS1	Centrosome	Centriole assembly	N/A	N/A	N/A	[100]
HsCEN3	MPS1	Centrosome	Inhibition of centrosome duplication	N/A	N/A	N/A	[101]
CDC31	MPS3p	SPB	SPB duplication	N/A	N/A	N/A	[102]
HsCEN2	CP110	Centrosome	Cytokinesis	N/A	N/A	N/A	[103]
HsCEN2	CDC25B	Centrosome Cytoplasm	Centrosome integrity	N/A	N/A	N/A	[104,105]
HsCEN2	Gelectin-3	Centrosome	N/A	N/A	N/A	N/A	[106]
CDC31	KIC1p	N/A	Cell integrity/ morphogenesis	N/A	N/A	N/A	[107,108]
CDC31	26S proteasome	Cytoplasm/ proteasome	Protein degradation	N/A	N/A	N/A	[28]
CDC31	cytosolic and mitochondrial factors	Mitochondria	Energy metabolism	N/A	N/A	N/A	[109]
CDC31	VPS13	N/A	TGN (trans-Golgi network) –PVC (prevacuolar compartment) transport and TGN homotypic fusion	N/A	N/A	N/A	[110,111]
AtCEN1	Tonneau1	Cytoskeleton	microtubule centers organization	N/A	N/A	N/A	[1]

^a^ K_a_ = equilibrium association constant; K_d_ = dissociation constant. K_d_ is the inverse of the equilibrium association constant, K_a_, (i.e., K_d_ = 1/K_a_). ^b^ N/A not available. ^c^ The repeats (R) in SFI1 are numbered as they appear in the protein sequence.

## 4. Centrin–Peptide Complexes

While in some cases the function of centrins has been linked with a specific biological process, overall, there is still limited knowledge about their structural properties and centrin–target interactions.

### 4.1. Centrins and Nucleotide Excision Repair

The autosomal recessive disorder xeroderma pigmentosum (XP), in humans, is associated with defects in nucleotide excision repair (NER). NER consists of two processes: transcription-coupled NER that removes transcription-blocking lesions and global genome NER that is initiated by a complex composed of XPC, HsCEN2, and RAD23B [27]. In this case, centrin interacts with XPC [27] and increases the specificity of the XPC/RAD23B complex for DNA lesions, thus playing a direct role in NER [17]. Investigations on Arabidopsis AtCEN2 have confirmed that centrin has a crucial role in NER [18,112]. AtCEN2, in fact, interacts with AtRAD4, the Arabidopsis homolog of human XPC, in a Ca^2+^ dependent manner [18,22] (Table 2). In yeast, centrin CDC31 is also a partner in a complex that similarly involves RAD4/RAD23 [28] and the C-terminal domain of CDC31 binds RAD4. Thus, a common regulatory mechanism may be present in eukaryotes.

**Figure 2:**
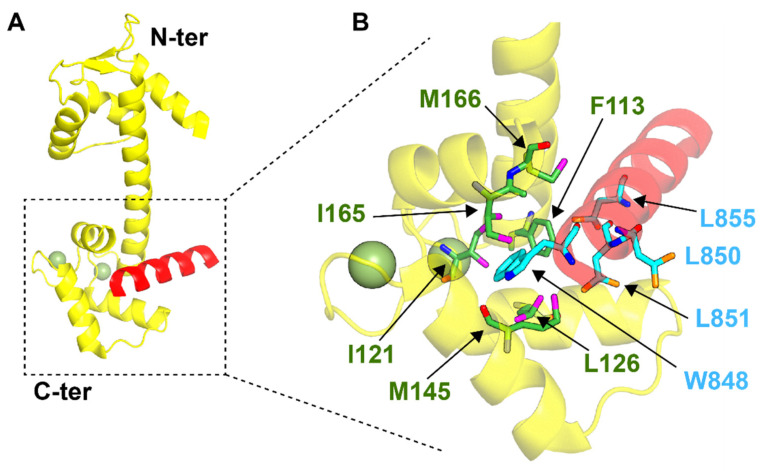
Crystal structure of the complex HsCEN2/P17-XPC (PDB: 2OBH). (**A**) Global view of the complex. The centrin molecule is represented in yellow and the P17-XPC peptide is shown in red. The Ca^2+^ ions at the C-terminal binding sites of the centrin molecule are represented as smudge green spheres. (**B**) Magnification of the binding site of HsCEN2. Key residues, defining the interface of interaction, are reported as green (HsCEN2) and cyan (P17-XPC) residues.

The contact between HsCEN2 and XPC occurs through a high affinity binding site that is located between N847 and R863 in XPC [76] (Table 2). A 17 amino acids peptide corresponding to this binding (P17-XPC) increases its affinity for HsCEN2 by 20-fold when Ca^2+^ is present [76]. The X-ray structure has shown that the peptide fits well in the hydrophobic pocket located in the C-terminal domain of centrin (PDB: 2OBH) (Figure 2). W848, L851, and L855 of P17-XPC seem to be the major residues involved in binding [49]. W848 is bound tightly in the cavity and residues F113, M145, and I165 of human centrin all form hydrophobic and polar interactions with the indole moiety. There is also a crucial bond between the guanidium group of R858 and R860 of the peptide and centrin residues E105 and E132, respectively. The complex is further stabilized by hydrophobic contacts involving L851 and L132 [49]. Interestingly, W848 and L851 in P17-XPC superpose with the equivalent residues in SFI1 [70] and KAR1 [79], while in the case of L855, the divergences are more evident.

### 4.2. SFI1 and Centrosome Duplication

The protein SFI1 is found in the centrosome of most eukaryotes and is involved in assembly of the mitotic spindle and progression of the G2–M transition of the cell cycle [113]. Pull-down experiments [74] showed that SFI1 has the ability to interact with HsCEN2 in humans and CDC31 in yeast, and SFI1 and CDC31 mutants exhibit defects in SPB duplication in yeasts [74]. Of note, in addition to the SPB duplication, CDC31 was found to be required for meiotic SPB remodeling, thus revealing novel functions of this protein. However, how CDC31 is involved in this process remains to be elucidated [114].

SFI1 from *S. cerevisiae* and humans can bind up to ~20 and 25 molecules of centrin, respectively, using conserved binding sites that are repeated in the central α-helical portion of SFI1; these consensus repeats are AX_7_LLX_3_F/LX_2_W [74], and each repeat can bind one molecule of centrin (Table 2). HsSFI1 has regular 10 amino acid gaps between each repeat; in contrast, ScSFI1 has gaps that range from 0 to 12 amino acids.

**Figure 3:**
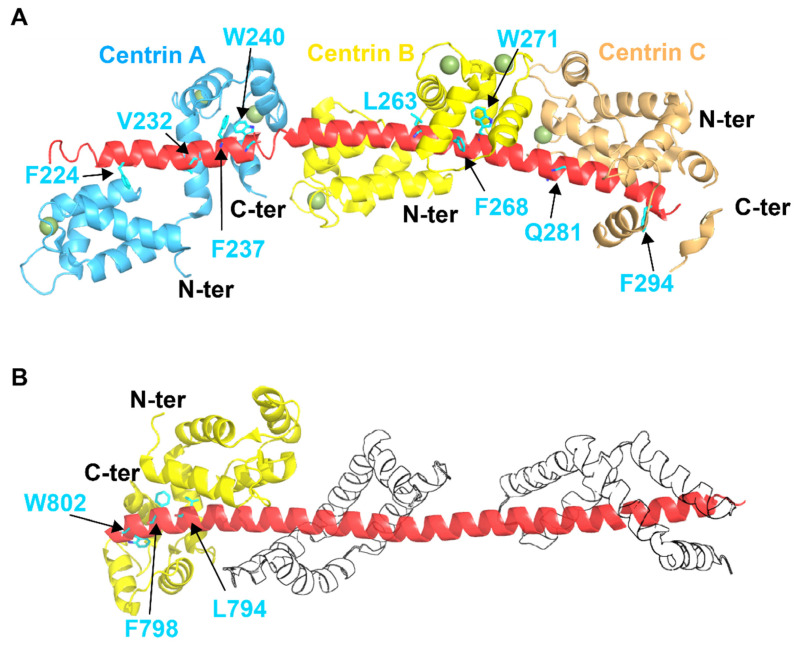
Crystal structures of (**A**) the complex between SFI1 and CDC31 molecules and (**B**) the complex between SAC3, SUS1, and CDC31. (**A**) Crystal structure of three yeast centrins CDC31 (light blue, yellow and orange) bound to SFI1 (PDB: 2DOQ). Ca^2+^ ions are indicated by smudge green spheres. The anchoring residues for the interaction of SFI1 with CDC31 are indicated in cyan. (**B**) 3D structure of the complex CDC31 (yellow), SAC3 (red) and SUS1 (transparent) (PDB: 3FWC). The key residues constituting the large hydrophobic surface for the interaction of SAC3 with CDC31 are indicated in cyan.

The crystal structure of CDC31 bound with two or three repeats of ScSFI1 reveals that the centrins are wrapped around long α-helix in ScSFI1 (PDB: 2DOQ) (Figure 3A) [70]. In CDC31, both the C- and N-terminal domains can bind ScSFI1. The C-lobe in CDC31 has an open conformation, while the N-terminal domain has a closed conformation [70]. The C-terminal domain of centrin mostly interacts with the C-terminus of the ScSFI1 repeats, analogously to the interactions between the C-lobe of *Chlamydomonas* centrin and KAR1 (PDB: 1OQP) (see below) [79]. Ca^2+^ does not seem to be needed for the binding between centrin and ScSFI1 given that the addition of the ion does not affect the conformation of centrin, at least when it is complexed with ScSFI1. The structure has also documented the presence of several centrin–centrin interactions, for the most part involving the C-terminal in a centrin with the N-terminal of another centrin. Such interactions suggest that centrin–centrin interactions might allow for the formation of filaments that are stabilized by further interactions with ScSFI1.

The structure of the complex between the C-terminal of HsCEN2 (T94–Y172) with the peptide R17-SFI1 has been resolved by NMR (PDB: 2K2I) [78,90]. Differently from the complex of yeast centrin with ScSFI1, in human centrin, the N-terminal domain has no substantial function in binding to HsSFI1. The W residue of SFI1 peptide lies within a hydrophobic cavity in which the centrin F113 residue is located, as occurs for XPC. The structure further revealed that the affinity of SFI1 peptide is decreased compared to that of P17-XPC because of a helix dipole inversion. Moreover, when compared with the NMR structure of the C-terminal domain of HsCEN2 and the P17-XPC peptide (PDB: 2A4J) [77], the HsCEN2 residue E148 appears to differentiate between XPC and SFI1 [115].

### 4.3. Centrins and mRNA Export

The TREX-2 complex plays a key role in transcription and mRNA nuclear export [24,93,116]. In *S. cerevisiae*, TREX-2 has five subunits (SAC3, THP1, CDC31, SUS1, and SEM1) [116]. SAC3 serves as the core scaffold for assembly with the other subunits. Fischer et al. reported that the C-terminal domain (CID) of SAC3 binds both CDC31 and SUS1 [24]. Comparison of the sequence of SAC3 CID with other peptides that have the ability to bind CDC31 advocated that the binding site is likely between amino acid residues 795 and 813. This sequence has several features that are shared with other CDC31 binding motifs [70,74]. In yeast, residues 795–813 of SAC3 tend to copurify with CDC31 [20], while their removal leads to a loss of the ability to bind CDC31 [20].

The interactions between CDC31, SUS1, and SAC3 have been studied by crystallography using SAC3 residues 723–805 in combination with CDC31 and SUS1 (PDB: 3FWC). (Figure 3B) [20]. In this complex, SAC3 exists as a long α helix that binds one molecule of CDC31 and two molecules of SUS1. The interaction between SAC3 and CDC31 primarily involves EF-3 and EF-4, which are located in the C-terminal domain of CDC31 [70]. The N-domain of CDC31 adopts a closed conformation, while the C-terminal domain has an open conformation [70]. Residues in the C-terminal domain of CDC31 are able to form a sort of hydrophobic margin with SAC3, which is similar to that seen in the interaction with SFI1 (PDB: 2DOQ) [70] and to those between *Chlamydomonas* centrin and KAR1 (PDB: 1OQP) (see below) [79], even if in SAC3 the helix has an opposite orientation [20]. W802 of SAC3 appears to have a primary role in this interaction and is nestled in a hydrophobic pocket in the C-terminal of CDC31, which is made by F105, M137, I138, F141, I149, and I157. Even if the CDC31-binding motifs in SAC3 (~24-residues) are shorter than those in the CDC31-binding region of SFI1 (~33 residues), both consensus CDC31 binding motifs have several features in common. The main difference between the binding sites in SFI1 and SAC3 is that the residues involved in binding the N-terminal domain of CDC31 are absent in SAC3 [108].

It remains unclear if the binding of SUS1 and CDC31 to the C-terminal domain of SAC3 is constitutively active or regulated. It would appear that CDC31 is regulated by changes in cellular levels of Ca^2+^. However, similar to the CDC31-SFI1 interaction [70], variations in Ca^2+^ levels did not influence the interaction between CDC31 with SAC3 [20]. In vitro investigations between CDC31 and a peptide containing the centrin binding motif in SAC3 established that the formation of this complex is only slightly affected by Ca^2+^. In fact, the K_d_ for SAC3 peptide binding to CDC31 was ~45 nM with Ca^2+^ compared to ~677 nM without [19].

The existence of SUS1, SAC3, and CDC31 homologs in other species raises the possibility that the C-terminal domain complex is conserved [92,95,117]. Based on the yeast structure, a putative motif in human SAC3/GANP was found that can bind to ENY2, the human SUS1 homolog, at least in vitro [20]. TREX-2 was recently identified in *A. thaliana* and is composed of the five proteins SAC3B, SAC3A, THP1, CEN1, and CEN2 [95]. Similar to SAC3 from yeast, SAC3B from Arabidopsis interacts with AtCEN2 [95]. The presence of a centrin-binding site in the C-terminal of SAC3B has been reported and its interaction characterized through spectroscopic and calorimetric approaches [21]. Of note, AtCEN2 can bind to SAC3B through the C-terminal domain independently of a stimulus involving Ca^2+^ [21].

### 4.4. Centrins and KAR1

KAR1 is an important constituent of the SPB in yeast and is needed for cell integrity [80]. Mutation of the KAR1 gene blocks SPB duplication at an early stage, leading to an enlarged SPB [80]. CDC31 binds to the central portion of KAR1 (residues 237–255, Table 2), which is localized to the half bridge of the SPB [80,118]. In addition, the corresponding 19-mer peptide, which contains the conserved 1-4-8 triad, can bind with high affinity in a Ca^2+^-dependent manner to CDC31 [97]. Creascu et al. reported that in the presence of Ca^2+^ the affinity for KAR1 and CDC31 is at least 50-fold higher than in its absence [19]. Mutational and biophysical analyses have documented that KAR1 interacts with CDC31 via the C-terminal domain [66,97].

**Figure 4:**
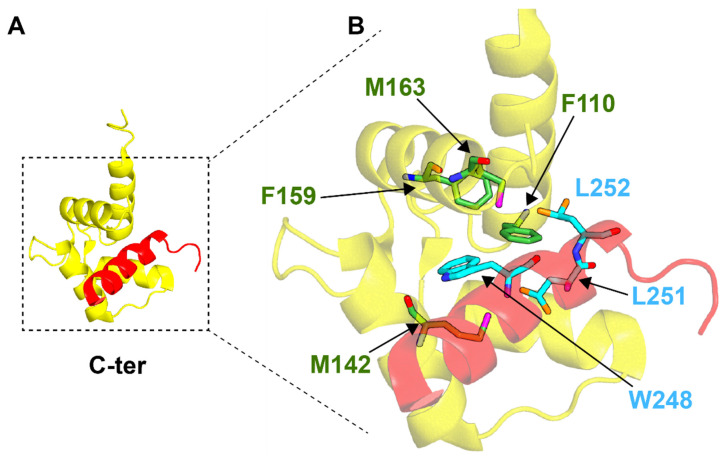
NMR structure of the complex between the C-terminal domain of CrCEN and the KAR1 peptide (PDB: 1OQP). (**A**) The centrin molecule is represented in yellow and the KAR1 peptide is shown in red. (**B**) Magnification of the binding site of CrCEN. Key residues, defining the interface of interaction, are reported as green (CrCEN) and cyan (KAR1 peptide) residues.

At present, there is no structure of the CDC31–KAR1 complex, although the structure of the Ca^2+^-activated C-terminal domain of *Chlamydomonas reinhardtii* centrin (CrCEN-C) complexed with the KAR1 peptide has been solved by NMR (PDB: 1OQP) (Figure 4) [79]. In this case, the protein complex is stabilized through specific interactions between three hydrophobic residues, namely W248, L251 and L252, and CrCEN-C in addition to electrostatic interactions between the basic peptide and the acidic binding site in CrCEN-C. All these residues reside in a hydrophobic cavity located on the CrCEN-C protein, and W248 is completely emersed in the deepest pocket. The protein–peptide interface is stabilized by two methionine residues (M142 and M63) and two phenylalanine residues (F110 and F159) in CrCEN-C. The greater affinity for Ca^2+^ by CrCEN-C when the KAR1 peptide is present has been explained by stabilization of the open conformation of CrCEN-C by the peptide.

As shown by mutational studies and direct measurements of binding affinity [64], in contrast to CrCEN-C, CrCEN-N does not have substantial binding affinity for KAR1 peptide, suggesting that they have different cellular targets [64,66].

## 5. Conclusions and Future Directions

Centrins are clearly crucial components of multiple signaling pathways in eukaryotic organisms, but there are still significant gaps in knowledge.

Herein, we surveyed structural properties of centrins and binding characteristics of centrin target proteins to shed light on the molecular mechanisms at the basis of the various functions of centrins and to obtain a more defined picture of how centrins contribute to the complexity of the Ca^2+^ signaling cascade.

The available knowledge suggests that centrins have dynamic roles and binding of Ca^2+^ is not always essential for interaction with their intracellular targets. Variations in EF-hands are predicted to relevantly contribute to the functional versatility of centrins and the differential Ca^2+^ affinities among centrins, some prebound to targets, others not, contribute to a system with considerable flexibility in responding to Ca^2+^ signals. Moreover, post-translational modifications also regulate the activity and subcellular localizations of centrins. All these regulatory mechanisms may permit organisms with comparatively fewer centrins to realize a functional diversity that is similar to organisms with a much larger number of centrins.

Notwithstanding, there is the need to gain more knowledge about the different regulatory conditions of centrins and place them in a functional context. Emerging high resolution imaging methods and proteomics approaches dedicated to deciphering protein complexes should help to understand how centrin proteins can act at the crossroad of various signaling pathway by binding such a diverse collection of proteins. Moreover, greater attention should be given to the ability of centrins to work as bridging molecules between proteins, besides their function in conveying the Ca^2+^ signal to specific target proteins.

As the structural and functional properties of centrins from other organisms become better understood, this will provide the basis for interpreting the complex Ca^2+^-based signaling system. This may also help to define the evolutionary pressures that led to conservation of centrinsand will also lead to new insights into the molecular evolution of Ca^2+^ binding proteins.

## References

[1] Azimzadeh J., Nacry P., Christodoulidou A., Drevensek S., Camilleri C., Amiour N., Parcy F., Pastuglia M., Bouchez D. (2008). Arabidopsis TONNEAU1 proteins are essential for preprophase band formation and interact with centrin. Plant Cell.

[2] Del Vecchio A.J., Harper J.D.I., Vaughn K.C., Baron A.T., Salisbury J.L., Overall R.L. (1997). Centrin homologues in higher plants are prominently associated with the developing cell plate. Protoplasma.

[3] Brugerolle G., Bricheux G., Coffe G. (2000). Centrin protein and genes in Trichomonas vaginalis and close relatives. J. Eukaryot. Microbiol..

[4] Salisbury J.L., Baron A., Surek B., Melkonian M. (1984). Striated flagellar roots: Isolation and partial characterization of a calcium-modulated contractile organelle. J. Cell Biol..

[5] Errabolu R., Sanders M.A., Salisbury J.L. (1994). Cloning of a cDNA encoding human centrin, an EF-hand protein of centrosomes and mitotic spindle poles. J. Cell Sci..

[6] Lee V.D., Huang B. (1993). Molecular cloning and centrosomal localization of human caltractin. Proc. Natl. Acad. Sci. USA.

[7] Ogawa K., Shimizu T. (1993). cDNA sequence for mouse caltractin. Biochim. Biophys. Acta.

[8] Spang A., Courtney I., Fackler U., Matzner M., Schiebel E. (1993). The calcium-binding protein cell division cycle 31 of Saccharomyces cerevisiae is a component of the half bridge of the spindle pole body. J. Cell Biol..

[9] Baum P., Furlong C., Byers B. (1986). Yeast gene required for spindle pole body duplication: Homology of its product with Ca^2+^-binding proteins. Proc. Natl. Acad. Sci. USA.

[10] Huang B., Mengersen A., Lee V.D. (1988). Molecular cloning of cDNA for caltractin, a basal body-associated Ca^2+^-binding protein: Homology in its protein sequence with calmodulin and the yeast CDC31 gene product. J. Cell Biol..

[11] Madeddu L., Klotz C., Le Caer J.P., Beisson J. (1996). Characterization of centrin genes in Paramecium. Eur. J. Biochem..

[12] Middendorp S., Paoletti A., Schiebel E., Bornens M. (1997). Identification of a new mammalian centrin gene, more closely related to Saccharomyces cerevisiae CDC31 gene. Proc. Natl. Acad. Sci. USA.

[13] Yaqin Z., Jiuying F., Aihua L., Binsheng Y. (2009). The characterization for the binding of calcium and terbium to Euplotes octocarinatus centrin. Spectrochim. Acta Part A Mol. Biomol. Spectrosc..

[14] Shan F., Ye K., Zhang J., Liao S., Zhang X., Xu C., Tu X. (2018). Solution structure of TbCentrin4 from Trypanosoma brucei and its interactions with Ca^2+^ and other centrins. Biochem. J..

[15] Hu K., Johnson J., Florens L., Fraunholz M., Suravajjala S., DiLullo C., Yates J., Roos D.S., Murray J.M. (2006). Cytoskeletal components of an invasion machine—The apical complex of Toxoplasma gondii. PLoS Pathog..

[16] Paoletti A., Moudjou M., Paintrand M., Salisbury J.L., Bornens M. (1996). Most of centrin in animal cells is not centrosome-associated and centrosomal centrin is confined to the distal lumen of centrioles. J. Cell Sci..

[17] Nishi R., Okuda Y., Watanabe E., Mori T., Iwai S., Masutani C., Sugasawa K., Hanaoka F. (2005). Centrin 2 stimulates nucleotide excision repair by interacting with xeroderma pigmentosum group C protein. Mol. Cell. Biol..

[18] Liang L., Flury S., Kalck V., Hohn B., Molinier J. (2006). CENTRIN2 interacts with the Arabidopsis homolog of the human XPC protein (AtRAD4) and contributes to efficient synthesis-dependent repair of bulky DNA lesions. Plant Mol. Biol..

[19] Miron S., Durand D., Chilom C., Perez J., Craescu C.T. (2011). Binding of calcium, magnesium, and target peptides to Cdc31, the centrin of yeast Saccharomyces cerevisiae. Biochemistry.

[20] Jani D., Lutz S., Marshall N.J., Fischer T., Kohler A., Ellisdon A.M., Hurt E., Stewart M. (2009). Sus1, Cdc31, and the Sac3 CID region form a conserved interaction platform that promotes nuclear pore association and mRNA export. Mol. Cell.

[21] Pedretti M., Conter C., Dominici P., Astegno A. (2020). SAC3B is a target of CML19, the centrin 2 of Arabidopsis thaliana. Biochem. J..

[22] La Verde V., Trande M., D’Onofrio M., Dominici P., Astegno A. (2018). Binding of calcium and target peptide to calmodulin-like protein CML19, the centrin 2 of Arabidopsis thaliana. Int. J. Biol. Macromol..

[23] Giessl A., Pulvermüller A., Trojan P., Park J.H., Choe H.-W., Ernst O.P., Hofmann K.P., Wolfrum U. (2004). Differential Expression and Interaction with the Visual G-protein Transducin of Centrin Isoforms in Mammalian Photoreceptor Cells. J. Biol. Chem..

[24] Fischer T., Rodríguez-Navarro S., Pereira G., Rácz A., Schiebel E., Hurt E. (2004). Yeast centrin Cdc31 is linked to the nuclear mRNA export machinery. Nat. Cell Biol..

[25] Resendes K.K., Rasala B.A., Forbes D.J. (2008). Centrin 2 localizes to the vertebrate nuclear pore and plays a role in mRNA and protein export. Mol. Cell. Biol..

[26] Zhang Y., He C.Y. (2012). Centrins in unicellular organisms: Functional diversity and specialization. Protoplasma.

[27] Araki M., Masutani C., Takemura M., Uchida A., Sugasawa K., Kondoh J., Ohkuma Y., Hanaoka F. (2001). Centrosome protein centrin 2/caltractin 1 is part of the xeroderma pigmentosum group C complex that initiates global genome nucleotide excision repair. J. Biol. Chem..

[28] Chen L., Madura K. (2008). Centrin/Cdc31 is a novel regulator of protein degradation. Mol. Cell. Biol..

[29] Grecu D., Assairi L. (2014). CK2 phosphorylation of human centrins 1 and 2 regulates their binding to the DNA repair protein XPC, the centrosomal protein Sfi1 and the phototransduction protein transducin β. FEBS Open Bio.

[30] Gavet O., Alvarez C., Gaspar P., Bornens M. (2003). Centrin4p, a novel mammalian centrin specifically expressed in ciliated cells. Mol. Biol. Cell.

[31] Hart P.E., Glantz J.N., Orth J.D., Poynter G.M., Salisbury J.L. (1999). Testis-specific murine centrin, Cetn1: Genomic characterization and evidence for retroposition of a gene encoding a centrosome protein. Genomics.

[32] Friedberg F. (2006). Centrin isoforms in mammals. Relation to calmodulin. Mol. Biol. Rep..

[33] Aubusson-Fleury A., Balavoine G., Lemullois M., Bouhouche K., Beisson J., Koll F. (2017). Centrin diversity and basal body patterning across evolution: New insights from Paramecium. Biol. Open.

[34] Mahajan B., Selvapandiyan A., Gerald N.J., Majam V., Zheng H., Wickramarachchi T., Tiwari J., Fujioka H., Moch J.K., Kumar N. (2008). Centrins, cell cycle regulation proteins in human malaria parasite Plasmodium falciparum. J. Biol. Chem..

[35] He C.Y., Pypaert M., Warren G. (2005). Golgi duplication in Trypanosoma brucei requires Centrin2. Science.

[36] Berriman M., Ghedin E., Hertz-Fowler C., Blandin G., Renauld H., Bartholomeu D.C., Lennard N.J., Caler E., Hamlin N.E., Haas B. (2005). The genome of the African trypanosome Trypanosoma brucei. Science.

[37] Bornens M., Azimzadeh J. (2007). Origin and Evolution of the Centrosome. Eukaryotic Membranes and Cytoskeleton: Origins and Evolution.

[38] Ruiz F., Garreau de Loubresse N., Klotz C., Beisson J., Koll F. (2005). Centrin deficiency in Paramecium affects the geometry of basal-body duplication. Curr. Biol..

[39] Gonda K., Yoshida A., Oami K., Takahashi M. (2004). Centrin is essential for the activity of the ciliary reversal-coupled voltage-gated Ca^2+^ channels. Biochem. Biophys. Res. Commun..

[40] Gogendeau D., Klotz C., Arnaiz O., Malinowska A., Dadlez M., de Loubresse N.G., Ruiz F., Koll F., Beisson J. (2008). Functional diversification of centrins and cell morphological complexity. J. Cell Sci..

[41] Babu Y.S., Bugg C.E., Cook W.J. (1988). Structure of calmodulin refined at 2.2 A resolution. J. Mol. Biol..

[42] Kuboniwa H., Tjandra N., Grzesiek S., Ren H., Klee C.B., Bax A. (1995). Solution structure of calcium-free calmodulin. Nat. Struct. Biol..

[43] Thompson J.R., Ryan Z.C., Salisbury J.L., Kumar R. (2006). The Structure of the Human Centrin 2-Xeroderma Pigmentosum Group C Protein Complex. J. Biol. Chem..

[44] Gifford J.L., Walsh M.P., Vogel H.J. (2007). Structures and metal-ion-binding properties of the Ca^2+^-binding helix-loop-helix EF-hand motifs. Biochem. J..

[45] Trande M., Pedretti M., Bonza M.C., Di Matteo A., D’Onofrio M., Dominici P., Astegno A. (2019). Cation and peptide binding properties of CML7, a calmodulin-like protein from Arabidopsis thaliana. J. Inorg. Biochem..

[46] Bhattacharya D., Steinkötter J., Melkonian M. (1993). Molecular cloning and evolutionary analysis of the calcium-modulated contractile protein, centrin, in green algae and land plants. Plant Mol. Biol..

[47] de Castro E., Sigrist C.J., Gattiker A., Bulliard V., Langendijk-Genevaux P.S., Gasteiger E., Bairoch A., Hulo N. (2006). ScanProsite: Detection of PROSITE signature matches and ProRule-associated functional and structural residues in proteins. Nucleic Acids Res..

[48] Durussel I., Blouquit Y., Middendorp S., Craescu C.T., Cox J.A. (2000). Cation- and peptide-binding properties of human centrin 2. FEBS Lett..

[49] Charbonnier J.B., Renaud E., Miron S., Le Du M.H., Blouquit Y., Duchambon P., Christova P., Shosheva A., Rose T., Angulo J.F. (2007). Structural, thermodynamic, and cellular characterization of human centrin 2 interaction with xeroderma pigmentosum group C protein. J. Mol. Biol..

[50] Matei E., Miron S., Blouquit Y., Duchambon P., Durussel I., Cox J.A., Craescu C.T. (2003). C-terminal half of human centrin 2 behaves like a regulatory EF-hand domain. Biochemistry.

[51] Kim S.Y., Kim D.S., Hong J.E., Park J.H. (2017). Crystal structure of wild-type centrin 1 from Mus musculus occupied by Ca^2+^. Biochemistry.

[52] Radu L., Durussel I., Assairi L., Blouquit Y., Miron S., Cox J.A., Craescu C.T. (2010). Scherffelia dubia centrin exhibits a specific mechanism for Ca^2+^-controlled target binding. Biochemistry.

[53] Bombardi L., Pedretti M., Conter C., Dominici P., Astegno A. (2020). Distinct Calcium Binding and Structural Properties of Two Centrin Isoforms from Toxoplasma gondii. Biomolecules.

[54] Astegno A., Bonza M.C., Vallone R., La Verde V., D’Onofrio M., Luoni L., Molesini B., Dominici P. (2017). Arabidopsis calmodulin-like protein CML36 is a calcium (Ca^2+^) sensor that interacts with the plasma membrane Ca^2+^-ATPase isoform ACA8 and stimulates its activity. J. Biol. Chem..

[55] Vallone R., La Verde V., D’Onofrio M., Giorgetti A., Dominici P., Astegno A. (2016). Metal binding affinity and structural properties of calmodulin-like protein 14 from Arabidopsis thaliana. Protein Sci..

[56] Ogunrinde A., Munro K., Davidson A., Ubaid M., Snedden W.A. (2017). Arabidopsis Calmodulin-Like Proteins, CML15 and CML16 Possess Biochemical Properties Distinct from Calmodulin and Show Non-overlapping Tissue Expression Patterns. Front. Plant Sci..

[57] La Verde V., Dominici P., Astegno A. (2018). Towards Understanding Plant Calcium Signaling through Calmodulin-Like Proteins: A Biochemical and Structural Perspective. Int. J. Mol. Sci..

[58] Linse S., Helmersson A., Forsen S. (1991). Calcium binding to calmodulin and its globular domains. J. Biol. Chem..

[59] Astegno A., La Verde V., Marino V., Dell’Orco D., Dominici P. (2016). Biochemical and biophysical characterization of a plant calmodulin: Role of the N- and C-lobes in calcium binding, conformational change, and target interaction. Biochim. Biophys. Acta (BBA) Proteins Proteom..

[60] Wiech H., Geier B.M., Paschke T., Spang A., Grein K., Steinkotter J., Melkonian M., Schiebel E. (1996). Characterization of green alga, yeast, and human centrins. Specific subdomain features determine functional diversity. J. Biol. Chem..

[61] Tourbez M., Firanescu C., Yang A., Unipan L., Duchambon P., Blouquit Y., Craescu C.T. (2004). Calcium-dependent Self-assembly of Human Centrin 2. J. Biol. Chem..

[62] Yang A., Miron S., Duchambon P., Assairi L., Blouquit Y., Craescu C.T. (2006). The N-terminal domain of human centrin 2 has a closed structure, binds calcium with a very low affinity, and plays a role in the protein self-assembly. Biochemistry.

[63] Conter C., Bombardi L., Pedretti M., Favretto F., Di Matteo A., Dominici P., Astegno A. (2021). The interplay of self-assembly and target binding in centrin 1 from Toxoplasma gondii. Biochem. J..

[64] Veeraraghavan S., Fagan P.A., Hu H., Lee V., Harper J.F., Huang B., Chazin W.J. (2002). Structural independence of the two EF-hand domains of caltractin. J. Biol. Chem..

[65] Weber C., Lee V.D., Chazin W.J., Huang B. (1994). High level expression in Escherichia coli and characterization of the EF-hand calcium-binding protein caltractin. J. Biol. Chem..

[66] Hu H., Sheehan J.H., Chazin W.J. (2004). The Mode of Action of Centrin: Binding of Ca^2+^ and a peptide fragment of Kar1p to the C-terminal domain. J. Biol. Chem..

[67] Phanindranath R., Sudhakar D.V.S., Thangaraj K., Sharma Y. (2021). Conformational scanning of individual EF-hand motifs of calcium sensor protein centrin-1. Biochem. Biophys. Res. Commun..

[68] Phanindranath R., Sudhakar D.V., Sharma A.K., Thangaraj K., Sharma Y. (2016). Optimization of purification method and characterization of recombinant human Centrin-1. Protein Expr. Purif..

[69] Cox J.A., Tirone F., Durussel I., Firanescu C., Blouquit Y., Duchambon P., Craescu C.T. (2005). Calcium and magnesium binding to human centrin 3 and interaction with target peptides. Biochemistry.

[70] Li S., Sandercock A.M., Conduit P., Robinson C.V., Williams R.L., Kilmartin J.V. (2006). Structural role of Sfi1p–centrin filaments in budding yeast spindle pole body duplication. J. Cell Biol..

[71] Shan F., Yang X., Diwu Y., Ma H., Tu X. (2019). Trypanosoma brucei centrin5 is enriched in the flagellum and interacts with other centrins in a calcium-dependent manner. FEBS Open Bio.

[72] Wang Z.-J., Zhao Y.-Q., Ren L.-X., Li G.-T., Liang A.-H., Yang B.-S. (2007). Spectral study on the interaction of ciliate Euplotes octocarinatus centrin and metal ions. J. Photochem. Photobiol. A Chem..

[73] Camargo A.I., Wiggers H.J., Damalio J.C.P., Araujo A.P.U., Ribichich K.F., de Camargo P.C. (2013). Structural and thermodynamic studies of two centrin isoforms from Blastocladiella emersonii upon calcium binding. Biochim. Biophys. Acta (BBA) Proteins Proteom..

[74] Kilmartin J.V. (2003). Sfi1p has conserved centrin-binding sites and an essential function in budding yeast spindle pole body duplication. J. Cell Biol..

[75] Díaz Casas A., Chazin W.J., Pastrana-Ríos B. (2017). Prp40 Homolog A Is a Novel Centrin Target. Biophys. J..

[76] Popescu A., Miron S., Blouquit Y., Duchambon P., Christova P., Craescu C.T. (2003). Xeroderma pigmentosum group C protein possesses a high affinity binding site to human centrin 2 and calmodulin. J. Biol. Chem..

[77] Yang A., Miron S., Mouawad L., Duchambon P., Blouquit Y., Craescu C.T. (2006). Flexibility and Plasticity of Human Centrin 2 Binding to the Xeroderma Pigmentosum Group C Protein (XPC) from Nuclear Excision Repair. Biochemistry.

[78] Martinez-Sanz J., Kateb F., Assairi L., Blouquit Y., Bodenhausen G., Abergel D., Mouawad L., Craescu C.T. (2010). Structure, dynamics and thermodynamics of the human centrin 2/hSfi1 complex. J. Mol. Biol..

[79] Hu H., Chazin W.J. (2003). Unique features in the C-terminal domain provide caltractin with target specificity. J. Mol. Biol..

[80] Biggins S., Rose M.D. (1994). Direct interaction between yeast spindle pole body components: Kar1p is required for Cdc31p localization to the spindle pole body. J. Cell Biol..

[81] Liang Y., Pan J. (2013). Regulation of flagellar biogenesis by a calcium dependent protein kinase in Chlamydomonas reinhardtii. PLoS ONE.

[82] Lukasiewicz K.B., Greenwood T.M., Negron V.C., Bruzek A.K., Salisbury J.L., Lingle W.L. (2011). Control of centrin stability by Aurora A. PLoS ONE.

[83] Lingle W.L., Lutz W.H., Ingle J.N., Maihle N.J., Salisbury J.L. (1998). Centrosome hypertrophy in human breast tumors: Implications for genomic stability and cell polarity. Proc. Natl. Acad. Sci. USA.

[84] Trojan P., Krauss N., Choe H.W., Giessl A., Pulvermüller A., Wolfrum U. (2008). Centrins in retinal photoreceptor cells: Regulators in the connecting cilium. Prog. Retin. Eye Res..

[85] Meyn S.M., Seda C., Campbell M., Weiss K.L., Hu H., Pastrana-Rios B., Chazin W.J. (2006). The biochemical effect of Ser167 phosphorylation on Chlamydomonas reinhardtii centrin. Biochem. Biophys. Res. Commun..

[86] Sanoguet Z., Campbell M., Ramos S., Seda C., Moreno L.P., Pastrana-Rios B. (2006). Effects of Phosphorylation in Chlamydomonas Centrin Ser 167. Calcium Bind Proteins.

[87] Krasikova Y.S., Rechkunova N.I., Maltseva E.A., Craescu C.T., Petruseva I.O., Lavrik O.I. (2012). Influence of centrin 2 on the interaction of nucleotide excision repair factors with damaged DNA. Biochemistry..

[88] Nishi R., Sakai W., Tone D., Hanaoka F., Sugasawa K. (2013). Structure-function analysis of the EF-hand protein centrin-2 for its intracellular localization and nucleotide excision repair. Nucleic Acids Res..

[89] Zhao Y., Guo X., Yang B. (2019). Calcium-induced human centrin 1 self-assembly and double-regulating the binding with peptide R18-Sfi1p. Int. J. Biol. Macromol..

[90] Martinez-Sanz J., Yang A., Blouquit Y., Duchambon P., Assairi L., Craescu C.T. (2006). Binding of human centrin 2 to the centrosomal protein hSfi1. FEBS J.

[91] Rüthnick D., Vitale J., Neuner A., Schiebel E. (2021). The N-terminus of Sfi1 and yeast centrin Cdc31 provide the assembly site for a new spindle pole body. J. Cell Biol..

[92] Jani D., Lutz S., Hurt E., Laskey R.A., Stewart M., Wickramasinghe V.O. (2012). Functional and structural characterization of the mammalian TREX-2 complex that links transcription with nuclear messenger RNA export. Nucleic Acids Res..

[93] González-Aguilera C., Tous C., Gómez-González B., Huertas P., Luna R., Aguilera A. (2008). The THP1-SAC3-SUS1-CDC31 complex works in transcription elongation-mRNA export preventing RNA-mediated genome instability. Mol. Biol. Cell.

[94] Jani D., Valkov E., Stewart M. (2014). Structural basis for binding the TREX2 complex to nuclear pores, GAL1 localisation and mRNA export. Nucleic Acids Res..

[95] Lu Q., Tang X., Tian G., Wang F., Liu K., Nguyen V., Kohalmi S.E., Keller W.A., Tsang E.W.T., Harada J.J. (2010). Arabidopsis homolog of the yeast TREX-2 mRNA export complex: Components and anchoring nucleoporin. Plant J..

[96] Yang Y., La H., Tang K., Miki D., Yang L., Wang B., Duan C.-G., Nie W., Wang X., Wang S. (2017). SAC3B, a central component of the mRNA export complex TREX-2, is required for prevention of epigenetic gene silencing in Arabidopsis. Nucleic Acids Res..

[97] Geier B.M., Wiech H., Schiebel E. (1996). Binding of Centrins and Yeast Calmodulin to Synthetic Peptides Corresponding to Binding Sites in the Spindle Pole Body Components Kar1p and Spc110p*. J. Biol. Chem..

[98] Azimzadeh J., Hergert P., Delouvée A., Euteneuer U., Formstecher E., Khodjakov A., Bornens M. (2009). hPOC5 is a centrin-binding protein required for assembly of full-length centrioles. J. Cell Biol..

[99] Dantas T.J., Daly O.M., Conroy P.C., Tomas M., Wang Y., Lalor P., Dockery P., Ferrando-May E., Morrison C.G. (2013). Calcium-binding capacity of centrin2 is required for linear POC5 assembly but not for nucleotide excision repair. PLoS ONE.

[100] Yang C.-H., Kasbek C., Majumder S., Yusof A.M., Fisk H.A. (2010). Mps1 Phosphorylation Sites Regulate the Function of Centrin 2 in Centriole Assembly. Mol. Biol. Cell.

[101] Sawant D.B., Majumder S., Perkins J.L., Yang C.H., Eyers P.A., Fisk H.A. (2015). Centrin 3 is an inhibitor of centrosomal Mps1 and antagonizes centrin 2 function. Mol. Biol. Cell.

[102] Jaspersen S.L., Giddings T.H., Winey M. (2002). Mps3p is a novel component of the yeast spindle pole body that interacts with the yeast centrin homologue Cdc31p. J. Cell Biol..

[103] Tsang W.Y., Spektor A., Luciano D.J., Indjeian V.B., Chen Z., Salisbury J.L., Sánchez I., Dynlacht B.D. (2006). CP110 cooperates with two calcium-binding proteins to regulate cytokinesis and genome stability. Mol. Biol. Cell.

[104] Boutros R., Lorenzo C., Mondesert O., Jauneau A., Oakes V., Dozier C., Gabrielli B., Ducommun B. (2011). CDC25B associates with a centrin 2-containing complex and is involved in maintaining centrosome integrity. Biol. Cell.

[105] Boutros R., Mondesert O., Lorenzo C., Astuti P., McArthur G., Chircop M., Ducommun B., Gabrielli B. (2013). CDC25B overexpression stabilises centrin 2 and promotes the formation of excess centriolar foci. PLoS ONE.

[106] Koch A., Poirier F., Jacob R., Delacour D. (2010). Galectin-3, a novel centrosome-associated protein, required for epithelial morphogenesis. Mol. Biol. Cell.

[107] Sullivan D.S., Biggins S., Rose M.D. (1998). The yeast centrin, cdc31p, and the interacting protein kinase, Kic1p, are required for cell integrity. J. Cell Biol..

[108] Ivanovska I., Rose M.D. (2001). Fine structure analysis of the yeast centrin, Cdc31p, identifies residues specific for cell morphology and spindle pole body duplication. Genetics.

[109] Chen L., Bian S., Li H., Madura K. (2018). A role for Saccharomyces cerevisiae Centrin (Cdc31) in mitochondrial function and biogenesis. Mol. Microbiol..

[110] Myers M.D., Payne G.S. (2017). Vps13 and Cdc31/centrin: Puzzling partners in membrane traffic. J. Cell Biol..

[111] De M., Oleskie A.N., Ayyash M., Dutta S., Mancour L., Abazeed M.E., Brace E.J., Skiniotis G., Fuller R.S. (2017). The Vps13p–Cdc31p complex is directly required for TGN late endosome transport and TGN homotypic fusion. J. Cell Biol..

[112] Molinier J., Ramos C., Fritsch O., Hohn B. (2004). CENTRIN2 modulates homologous recombination and nucleotide excision repair in Arabidopsis. Plant Cell.

[113] Ma P., Winderickx J., Nauwelaers D., Dumortier F., De Doncker A., Thevelein J.M., Van Dijck P. (1999). Deletion of SFI1, a novel suppressor of partial Ras-cAMP pathway deficiency in the yeast Saccharomyces cerevisiae, causes G(2) arrest. Yeast.

[114] Ohta M., Sato M., Yamamoto M. (2012). Spindle pole body components are reorganized during fission yeast meiosis. Mol. Biol. Cell.

[115] Grecu D., Blouquit Y., Assairi L. (2014). The E144 residue of Scherffelia dubia centrin discriminates between the DNA repair protein XPC and the centrosomal protein Sfi1. FEBS Open Bio.

[116] Fischer T., Strässer K., Rácz A., Rodriguez-Navarro S., Oppizzi M., Ihrig P., Lechner J., Hurt E. (2002). The mRNA export machinery requires the novel Sac3p-Thp1p complex to dock at the nucleoplasmic entrance of the nuclear pores. EMBO J..

[117] Kurshakova M.M., Krasnov A.N., Kopytova D.V., Shidlovskii Y.V., Nikolenko J.V., Nabirochkina E.N., Spehner D., Schultz P., Tora L., Georgieva S.G. (2007). SAGA and a novel Drosophila export complex anchor efficient transcription and mRNA export to NPC. EMBO J..

[118] Spang A., Courtney I., Grein K., Matzner M., Schiebel E. (1995). The Cdc31p-binding protein Kar1p is a component of the half bridge of the yeast spindle pole body. J. Cell Biol..

